# How a Clinical Trial Unit can improve independent clinical research in rare tumors: the Italian Sarcoma Group experience

**DOI:** 10.1186/s13569-017-0068-4

**Published:** 2017-02-28

**Authors:** Emanuela Marchesi, Celeste Cagnazzo, Irene Quattrini, Martina Piccinni Leopardi, Chiara Villa, Giovanni Grignani, Lorenzo D’Ambrosio, Silvia Stacchiotti, Paolo Giovanni Casali, Piero Picci

**Affiliations:** 1 Italian Sarcoma Group, Istituto Ortopedico Rizzoli, Via Pupilli 1, 40136 Bologna, Italy; 2 Division of Medical Oncology, Candiolo Cancer Institute-FPO, IRCCS, Candiolo, Italy; 3 Chemotherapy Unit, Orthopedic Rizzoli Institute, Bologna, Italy; 4 Adult Mesenchymal Tumour and Rare Cancer Medical Oncology Unit, Fondazione IRCCS Istituto Nazionale Tumori, Milan, Italy; 5 Laboratory of Experimental Oncology, Orthopedic Rizzoli Institute, Bologna, Italy

## Abstract

**Background:**

The Italian Sarcoma Group (ISG) is a nonprofit group of professionals established in 1997 aimed to improve the quality of care and promote the independent research in sarcomas. The increased regulatory requirements, the chance to increase the number of trials with other cooperative groups and an interest from pharmaceutical companies in supporting independent research, generated the need of an internal service for research management.

**Methods and results:**

In 2010, ISG implemented in its organization a Clinical Trial Unit (CTU). The CTU was appointed to fully manage Clinical Trial Operations, to guarantee regulation compliance and provide a central support to the investigators, fostering a collaboration both at national and international level. In 2016 ISG promoted 25 studies in about 120 centers, with a fivefold increase in the last 5 years: 68% were interventional and 32% observational. Nine of the 17 interventional studies (52%) were supported by pharmaceutical companies, while 4 (24%) were funded by European Commission within specific projects on sarcomas and 4 (24%) were supported by the ISG itself.

**Conclusion:**

The contribution of ISG researchers to the international community was striking from the earliest years of the ISG creation. The challenges of the regulatory clinical research scenario, which imposes solid and hard-fast methodology with deep knowledge and expertise, highlighted the need to identify qualified and dedicated experts able to run and follow the multifaceted aspects of trials. Our analysis demonstrated how this model has led to a growth in competitiveness of the group. The collaboration between clinicians and CTU made possible to support the research with high scientific and ethical standards and to increase the number of trials, sites and overall enrolled patients. The reduced time for approvals, the continuous support to sites, the increased speed in data collection and analysis make the ISG research attractive for pharmaceutical industries, despite the problems that have characterized the independent research in the last years. The ability to fully manage and oversight Clinical Operations and the high quality of delivered services, have led the ISG to be recognized as a reliable partner and coordinator within the international sarcoma networks.

## Background

Sarcomas are rare tumors of mesenchymal origin, accounting for nearly 21 and <1% of all pediatric and adult solid malignant tumors, respectively [1].

Clinical trial research in disease with a low incidence like sarcomas (<6 per 100,000 people per year) is crucial to guarantee to the affected population the opportunity to access new treatments and benefit from medical and scientific progresses [2, 3].

However, in rare diseases, clinical research is challenging: few clinical trials are available for these populations and patients encounter difficulties in accessing specialized centers where the most innovative clinical trials are run [4].

In addition, conventional clinical trial designs are not always applicable and the statistical design needs to be modulated in order to make the studies feasible also in small populations [5–9].

The management of sarcomas poses several problems and the need for reference centers to share their experiences let to the creation of networks and consortia of specialists [10, 11].

From the late eighties the Italian reference centers specialized in sarcoma treatment, began to collaborate within some national research programs and participated to the development of investigator-initiated clinical trials [12, 13].

From this experience, they felt the need to share their expertise and joint together in an Italian research network aimed to improve the academic research and committed to try to provide answers to unmet clinical questions, especially in the extremely rare sarcomas, or for peculiar sarcoma subtypes.

Thus in 1997, the Italian Sarcoma Group (ISG) was founded and, legally recognized as a scientific independent non-profit association in 2002, begun to promote independent clinical trials.

From the time the European Clinical Trials Directive 2001/20/CE [14] was fully adopted in Italy, at the end of 2007, its effects resulted in an increased in strictness and complexity of trial activities and trial oversight procedures.

This regulation, the chance to increase the number of trials conducted within international consortia [15, 16] and a greater interest of pharmaceutical companies in supporting independent research, imposed the need to introduce in the ISG organization, a Clinical Trial Unit (CTU) exclusively dedicated to Clinical Trial Operations to serve as an operative focal point for national and collaborative trials in sarcomas.

## Methods

### Development of the Clinical Trial Unit

The proposal to implement a CTU was presented to the ISG Directive Executive Board in 2009 that granted its approval for the initial financial support.

The source of funds was covered by the fundraising activities of the group and by the internal overheads derived from previous supported studies The main CTU office was established at central level and, under the central coordination, two satellites offices were introduced in other institutions.

The assigned mission to CTU was to promote high quality standard of the group promoted research, to guarantee the compliance with regulation, to provide central support to participating sites, to facilitate collaboration between them and to collaborate with the other European sarcoma cooperative groups, involved in common international studies.

### Analysis

The impact of the CTU in ISG clinical research was assessed by comparing the number of clinical studies, the number of activated sites and of enrolled patients, the performance in data collection and the funds received to conduct the trials, in the time period from 2010 to the 3rd quarter of 2016.

The analysis included data of interventional, translational and observational studies as well as the educational activity promoted by the CTU.

## Results

The first step toward the CTU creation was to implement the Clinical Operation (ClinOp) services, within the ISG secretariat by part-time personnel with a previous specific expertise in the management of clinical trials in sarcomas.

Among the first services offered were those related to study monitoring (site initiation, monitoring, and close-out visits) and to the development of specific Clinical Trial Monitoring Standard Operative Procedures, (CTM-SOPs) to be applied to all the ISG clinical trials, in full compliance with the regulatory requirements.

From 2010 to 2012 the number of interventional clinical trials and centers completely managed by ISG ClinOp, increased respectively of 75% (from 4 to 7 studies) and 158% (from 26 to 67 activated sites).

As consequence to these first positive results an internal budget analysis was performed.

The recorded increase of funds (+48%) allowed ISG to introduce a fully independent CTU, able to cover its own expenses with their services.

CTU was then formally recognized by ISG and, from mid-2013, the ISG ClinOp were no longer part of the secretariat services, but started to be delivered by the independent CTU.

Responsibility of the CTU is to oversight the trial management for interventional and observational multi-sites clinical studies where ISG is sponsor or national coordinator within international consortia networks programs.

As reporte d in Table 1, the services offered by CTU to the group are focused on 5 main domains: scientific support, project management, safety and regulatory management, educational and CTU administrative management.

**Table 1:** Clinical Operation Services offered by ISG CTU

*Scientific support*
Protocol development
Support to the coordinator physician within the study board, for the implementation of the ethical aspects and safety requirements of research
Study report, publication and result dissemination
Support to the final study report, publication submission and dissemination of the study results
*Study project management*
Study budget plan and budget negotiation
Evaluation of study costs, budget plan development and budget negotiation with pharma companies supporters of the independent research or with partners within consortium projects
Study feasibility
Support to sites selection through site feasibility development within the study board
Regulatory documentation
Preparation of the regulatory documents, preparation and maintenance of the study binders in accordance with Good Clinical Practices and ISG Clinical Trial Standard Operating Procedure (SOP)
Regulatory submission
Submission to the Competent Authorities and Independent Review Boards (IRB) of the regulatory (protocols, patient’s information sheet and informed consents, Investigational Product information, safety information and all the others applicable documents)
Support activity in the preparation of the submission package for extra-Italian submission within international studies coordinated by ISG
Investigational Product (IP) activities
Management of the IP from labeling (if required) to supply and resupply activities
Database development
Support to the Case Report Form design
Investigators meetings
Support in the organization of the Investigators Meetings and logistics
Monitoring activities
Management and conduction, either remote and onsite, of the site activation, monitoring and close-out visits
Coordination of centralized activities
Development of procedures for centralized activities (samples management in case of centralized biological material analysis, centralized imaging review management)
Communication with the sites
Study state of art continuous monitoring
*Regulatory and safety management*
Safety regulatory activities conducted by the Pharmacovigilance Office
Communication of safety information (SUSAR, SAE line listing) to Competent Authorities and sites
Eudravigilance reporting activities
*Educational*
Protocol training activities
Training on GCP, regulatory and clinical operation
*Administrative*
Protocol budget plan
Site contract negotiation
External services contract negotiation

From the beginning of 2014 the CTU also introduced an independent pharmacovigilance office and developed Standard Operative Procedures (SOP) to cover all the safety related activities.

When the first ISG ClinOp services were implemented in 2010, 6 studies (4 interventional and 2 observational) were ongoing and were mainly managed by the coordinating investigators.

The trials promoted by ISG or in collaboration with other international sarcoma networks in mid-2016 were 25: 17 (68%) interventional and 8 (32%) observational (Table 2).

**Table 2:** Number and type of studies managed by the CTU

Year	Interventional	Observational
2010	4 (71%)	2 (29%)
2011	7 (78%)	2 (22%)
2012	7 (78%)	2 (22%)
2013	7 (78%)	2 (22%)
2014	12 (66%)	6 (34%)
2015	13 (68%)	6 (32%)
2016	17 (68%)	8 (32%)

52% (n = 9) of the interventional have financial support from pharmaceutical companies, while 24% (n = 4) received funds within sarcoma European Commission projects and 24% (n = 4) are supported by ISG itself (Table 3). Taking into account only the interventional studies, whereof regulatory requirements impose stringent quality standards, a great improvement in the number of managed clinical sites and patients has been recorded from 2010 to mid-2016, bringing the CTU to manage from 25 to 119 sites (+376%) and more than 800 active patients (+543%) with an mean increase, respectively, of 31 and 38% per year (Table 4).

**Table 3:** Funding sources for interventional studies

Year	Supported by pharma companies	Supported within EC sarcomas’ projects	Supported by ISG
2010	2/4 (50%)	0 (0%)	2 (50%)
2011	4/7 (57%)	1 (14%)	2 (29%)
2012	3/7 (43%)	1 (14%)	3 (43%)
2013	3/7 (43%)	1 (14%)	3 (43%)
2014	6/12 (50%)	3 (25%)	3 (25%)
2015	6/13 (46%)	4 (31%)	3 (23%)
2016	9/17 (52%)	4 (24%)	4 (24%)

**Table 4:** Number of interventional CT studies, sites and patients managed by the CTU

Year	Nr of ongoing interventional trials	Nr. of sites	New sites/year	Nr. of active patients	New active patients/year
2010	4	26	NE	127	NE
2011	7	48	23	207	80
2012	7	67	18	323	116
2013	7	74	8	503	180
2014	12	91	17	601	98
2015	13	107	16	797	196
2016	17	119	12	816	19

*NE* not evaluated

Also a speed up in the time of ethical approval and in first patient enrollment have been recorded: a reduced mean time for approval of 4 months (and of 2 months for the First Patient First Visit).

During its 6 years of activity the high quality standard of clinical trial management, together with the capability of providing a comprehensive service for the ClinOp, brought the Italian Sarcoma Group to increase its collaborations within international cooperative groups (from 0 to 9 cooperative studies).

The services offered by the CTU had also impact on the supported research activity, with a more than sevenfold increase of funds from 2010 to 2016, and a mean number of new contracts for supported trials, in the last 3 years, of 3.3 (Table 5).

**Table 5:** Number of new supported studies

Year	New supported studies
2010	0
2011	2
2012	1
2013	0
2014	5
2015	1
2016	4

On the other hand, the increase of research management activities brought the need to potentiate the CTU personnel that, in mid-2016 account for 2 full time units and 5 partially dedicated units from satellites.

The key effort of the CTU in facilitating the collaboration within consortia trials led ISG to join as partner or leader in several European consortia studies.

This also permitted a direct collaborations with other European sarcoma CTU through the sharing of SOP and of the best ClinOp practices.

All the results of the ISG clinical studies, oversight by the CTU, are object of international meeting presentations and are published in the most important peer reviewed oncology journals.

The CTU is also committed in promoting educational and training activities, like periodic courses, addressed not only to Clinical Research Coordinators (CRC) and to the Investigators interested in clinical trial regulation and research methodology. As part of the educational mission of the CTU, from 2013 ISG introduced in its annual meeting, a parallel section dedicated to clinical trial coordination where regulatory and breaking news from trial regulation and methodology are presented.

The personnel of the CTU also collaborate with national groups in research methodology education and is involved in research in the field of clinical trial regulations [17, 18].

## Discussion

In the last decade, ISG research network played a key role in the development of independent research in the sarcoma field, in Italy, promoting more than 30 clinical trials and managing over 100 sites with more than 800 patients. The number of clinical trials increased steadily from the CTU introduction, and the possibility to independently manage them, contributed to increase the number of consortia and pharmaceutical supported research.

This led to a significant annual income increase that resulted in a self-financing system where the CTU is able to provide to its own maintenance.

Reduced time for regulatory approvals, immediate and continuous support to sites, and speed up in data collection and analysis, are making the ISG clinical research attractive for pharmaceutical industries, despite the fact that independent research in this field has been extremely challenging in the last years.

Finally, the feedback received from the ISG investigators about the support provided by CTU is extremely positive and contributes to increase the network collaboration and involvement, not only in terms of recruitment and data quality, but also in terms of clinical trial coordination activities.

## Conclusion

Contribution of ISG researchers to the international community was considered from the earliest years of ISG creation.

The regulatory trial scenario requires a more solid and hard-fast methodology with depth knowledge and expertise. These challenges highlighted the need to identify expert and dedica ted professionals able to run, manage and follow the multifaceted aspects of innovative clinical trials.

The proactive collaboration between clinicians and CTU professionals made possible to support the increasing research activity with high scientific and ethical standards.

The introduction of the CTU within the ISG organization represented an important a chievement in the group’s research activity, by facilitating its conduction.

This allows the group to be competitive and be recognized as a reliable partner within the international sarcoma networks and to lead the conduction of consortia clinical trials.

## Abbreviations

ISG: Italian Sarcoma Group
CTU: Clinical Trial Unit
SOP: Standard Operative Procedure
CTM-SOP: Clinical Trial Monitoring Standard Operative Procedure
ClinOp: Clinical Operation (ClinOp)
FPFV: First Patient First Visit
FTE: full time equivalent
CRC: Clinical Research Coordinator
